# TOUCH® duo-mobile prosthesis in TMC osteoarthritis: two-year results and practical insights regarding key surgical steps and complication management

**DOI:** 10.1007/s00402-025-05926-5

**Published:** 2025-05-22

**Authors:** Pia-Elena Frey, Simeon C. Daeschler, Yusuf Naseri, Maximilian Franzen, Jan Sommer, Leila Harhaus, Benjamin Panzram

**Affiliations:** 1 Department of Orthopedics, University Hospital Heidelberg, Heidelberg, Germany; 2 BG Klinik Ludwigshafen, Department of Hand, Plastic, and Reconstructive Surgery, Burn Center at Heidelberg University, Ludwigshafen, Germany; 3 BG Klinikum Unfallkrankenhaus Berlin, Germany, Department of Hand-, Replantation- and Microsurgery and Chair of Hand-, Replantation- and Microsurgery, Charité Universitätsmedizin Berlin, Berlin, Germany

**Keywords:** Trapeziometacarpal joint prosthesis, Thumb carpometacarpal joint prosthesis, Osteoarthritis, Arthroplasty, Trapeziometacarpal joint arthritis, Dual mobility

## Abstract

**Introduction:**

The Touch® dual-mobility prosthesis is a well-established treatment for advanced trapeziometacarpal (TMC) joint osteoarthritis, offering an alternative to resection arthroplasty. Short-term studies suggest dual-mobility designs reduce dislocation and loosening compared to single-mobility prostheses. This retrospective study presents clinical outcomes after a mean follow-up of 24 months, focusing on revision surgery and providing insights about key surgical steps and the management of adverse events.

**Materials and methods:**

A total of 78 patients (88 prostheses) with TMC osteoarthritis underwent surgery between August 2019 and December 2023, performed by a single surgeon in a monocentric setting. Preoperative assessments and follow-ups were conducted at 6 weeks, 6 months, 12 months, and annually. Outcome measures included radiographic analysis, range of motion, grip/pinch strength, pain (NRS 1–10), and functional scores (qDASH, briefMHQ). Complications and revisions were recorded.

**Results:**

At a mean follow-up of 24 months (range 6–61 months), significant improvements in hand function, pain, and mobility were observed. Preoperative thumb MCP hyperextension (> 15° in 23 thumbs) was corrected to 6° on average, and thumb length was restored. Four patients (4.5%) required implant revision: two due to secondary cup dislocation after misplacement, two due to impingement. Seven secondary surgeries addressed wound healing disorders (*n* = 2) and secondary De Quervain tenosynovitis (*n* = 5). Kaplan-Meier analysis showed a 96% prosthesis survival rate at two years.

**Conclusions:**

The Touch® dual-mobility prosthesis demonstrates high effectiveness in improving pain, function, and thumb stability, with low revision rates. Restoration of thumb length and correction of hyperextension support its use as a reliable surgical option. These findings are consistent with existing literature suggesting superior long-term stability compared to single mobility implants. Identified surgical challenges highlight factors contributing to complications and emphasize intraoperative strategies to prevent revision.

## Introduction

Osteoarthritis (OA) of the trapeziometacarpal (TMC) joint is a common condition, particularly affecting individuals over 50 years of age [4, 50]. The prevalence of isolated radiographically diagnosed TMC arthritis is estimated to be 25–35% in the subpopulation of postmenopausal women and approximately 6% in men [4]. The overall prevalence of clinically relevant osteoarthritis appears to be approximately 1.4% in adults, with a peak of 5.3% in the subgroup of women aged 70–74 years [41]. Post-mortem studies report a prevalence of up to 75%, and Armstrong et al. found radiographic evidence of rhizarthrosis in 33% of women over the age of 45, a third of whom had symptoms [4, 5]. This condition significantly impairs the ability to perform everyday tasks due to pain, reduced range of motion, and diminished grip and pinch strength. As the disease progresses, these functional limitations increasingly affect the quality of life [43].

Primary treatment for TMC joint OA is non-surgical, typically including orthoses, physiotherapy, and analgesics​. However, surgical options are required when non-operative treatments fail. In Europe, TMC joint prostheses have emerged as a preferred alternative to resection arthroplasty (RA), the traditional treatment, in osteoarthritis stage II and III. Compared to RA, joint replacement offers several advantages, such as quicker recovery, preservation or restoration of thumb length, improved grip and pinch strength as well as the correction of hyperextension [13, 22, 26, 31, 45, 49].

The latest generation of dual-mobility implants is particularly promising, as it shows reduced rates of complications such as implant dislocation and loosening, which were prevalent with earlier single-mobility designs [10, 47]. Studies have recently shown a 10-year survival of 95% for the ARPE, 93% for the IVORY as well as 88% for the MAIA prosthesis [40, 47, 48]. However, comparable long-term data for the TOUCH® dual-mobility prosthesis remain unavailable.

Short- to mid-term results of the TOUCH® prosthesis in 107 patients show revision rates of 4.6% after a mean follow-up of 40 months [39] and an estimated 2-year survival rate of 96% in 130 patients in another study [29].

This study reports the clinical and functional outcomes of the TOUCH® prosthesis after a mean follow-up of 24 months, contributing to the growing evidence supporting dual-mobility prostheses in the treatment of TMC joint arthritis.

## Materials and methods

In this retrospective, monocentric cohort study, 78 patients (88 prostheses) were included, all of whom underwent surgery performed by a single surgeon between August 2019 and December 2023. The inclusion and exclusion criteria were meticulously applied to ensure the appropriate selection of patients. Ethical approval was granted by the local Ethics Committee, and informed consent was obtained from all participants or their legal guardians.

*Inclusion criteria*

- Primary osteoarthritis (Eaton and Littler stage II or III)
- Age ≥ 18 years
- Consent of the patient to participate in the study
- Capacity of the patient to consent

*Exclusion criteria*

- Age < 18 years
- Pregnancy
- Patient’s denial to participate in the study
- Concomitant scapho-trapezio-trapezoidal (STT) joint osteoarthritis (Eaton and Littler stage IV)

None of the patients had previously received intra-articular cortisone injections within 3 months prior to surgery. The indication for surgery was symptomatic primary TMC joint osteoarthritis refractory to non-surgical management. The surgical approach followed the manufacturer’s protocol, utilizing a dorsal approach to the joint.

Postoperatively, the thumb was immobilized with a bulky soft dressing for 7 to 10 days, followed by a thumb orthosis for one week and an additional two weeks overnight. During daytime, patients were encouraged to engage in free range of motion exercises without resistance. Full weight-bearing was permitted after six weeks. Follow-up visits were scheduled at 6 weeks, 6 months, 12 months, and annually thereafter.

The primary endpoints were implant survival and the assessment of adverse events. Hand function was evaluated as a secondary endpoint by clinical investigation as well as assessments for pain and functional scores. Satisfaction with the outcome was rated on a scale from 1 to 5 (1 = very dissatisfied, 2 = dissatisfied, 3 = neutral, 4 = satisfied, 5 = very satisfied). Range of motion (ROM) of the thumb in radial and palmar abduction was measured using standard goniometers. Thumb opposition was evaluated using the Kapandji score [25]. Pain was assessed using a numeric rating scale (NRS) that ranged from 1 (indicating no pain) to 10 (representing the worst imaginable pain) at rest and under load. Grip and pinch strength were assessed using a Jamar dynamometer on level two and pinch gauge (Jamar Technologies Inc. in Clifton, NJ, USA). The Quick Disabilities of the Arm, Shoulder, and Hand (QuickDASH) questionnaire, and the Brief Michigan Hand Questionnaire (MHQ) were used to determine hand function [7, 11, 35].

Radiographic assessments were performed preoperatively, intraoperatively and during follow-up at 6 weeks, 6 months, 12 months, and annually thereafter. Standard posterior-anterior (PA) and lateral views were used to evaluate the first metacarpophalangeal (MCP 1) hyperextension deformity as well as the stage of osteoarthritis according to the Eaton and Littler classification on preoperative radiographs. The degree of MCP1 hyperextension was assessed on lateral radiographs in resting position by measuring the angle between the longitudinal axes of the first metacarpal and proximal phalanx. Postoperative radiographs were analyzed for residual MCP 1 hyperextension, ossifications, dislocation and loosening as well as impingement. Post-operative thumb length restoration was assessed by measuring the mean first metacarpal-trapezium length (M1) in posterior-anterior views. The radiographs were analyzed by at least two researchers (surgeons who perform the surgery), including an examiner who was not involved in the treatment of the patients.

The Touch® prosthesis (KeriMedical, Les Acacias, Switzerland) is a dual-mobility implant designed for TMC joint arthroplasty. The prosthesis design with its polyethylene-on-metal tribological pairing has been extensively described in several prior publications [21, 22].

Descriptive statistics were used to analyze the results. Unless otherwise stated, means and 95% confidence intervals (CI) or ranges were calculated. Implant survival rates were estimated using the Kaplan-Meier method for the endpoint revision surgery with replacement or removal of at least one implant component. Statistical significance was assumed if the p-value was below 0.05. All values were calculated and visualized using GraphPad Prism 10 for Windows 64-bit, version 10.2.3 (403), 21 April 2024.

Differences before and after therapy were analyzed using a t-test for continuous data and the Wilcoxon signed-rank test for scores.

## Results

The mean follow-up was 24 months with a minimum follow-up of 6 months (range: 6–61 months). In total, 55 women and 23 men (sex assigned at birth) with 88 prostheses were included in this study. In 10 patients, the Touch® prosthesis was implanted in both hands. The majority of the patients (70.5%) was female. The mean age at surgery was 59 years (range: 42–77 years). Dominance of the right hand was observed in 92.9% of the patients, and 60.2% of the operated joints were left hands.

### Survival

The primary endpoint of this study was implant survival. Implant failure was defined as an adverse event leading to revision surgery with replacement or removal of at least one implant component or resulting in secondary trapeziectomy. Replacement of components as part of a wound revision was not considered as a revision but a secondary surgery. If the cause of complaints during follow-up visits was unclear, CT imaging was performed to determine the presence of bone pathology not visible on conventional radiographs.

**Fig. 1:**
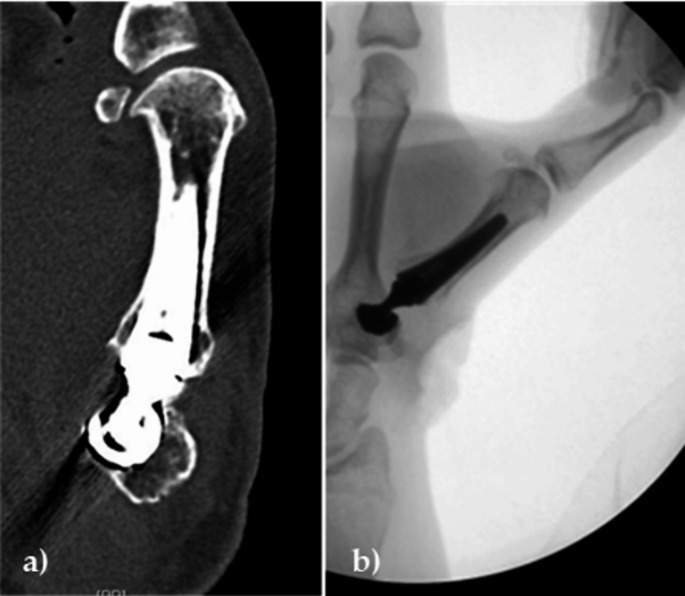
**a**) Radiographic imaging of a patient suffering from impingement between the neck and cup components, restricted movement shown in CT before revision surgery; **b**) Free movement of the prosthesis during intraoperative mobilization after replacement of the neck component

**Fig. 2:**
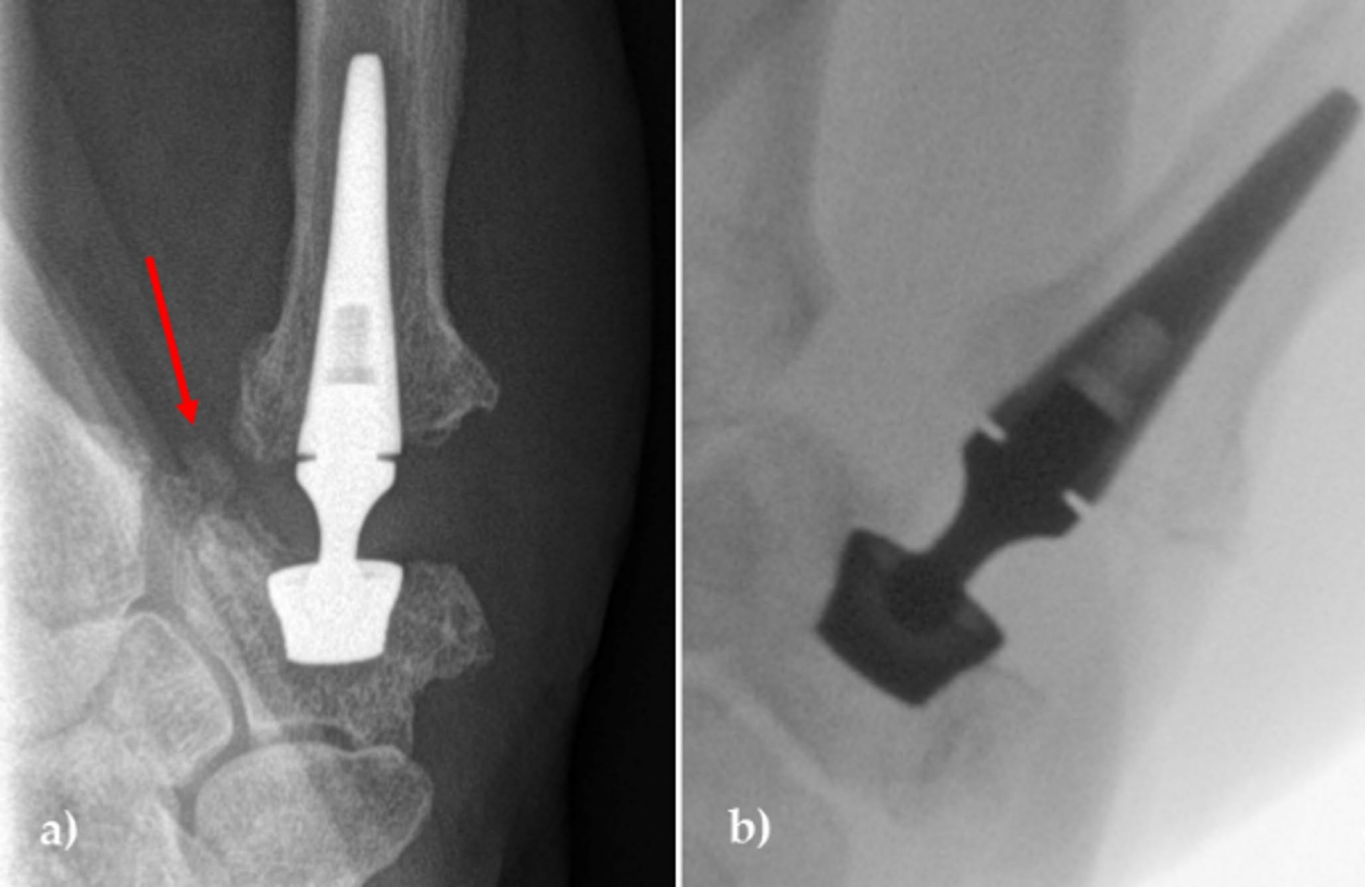
**a**) Osteophytes between the first and second metacarpals and the trapezium have been identified as the cause of impingement; **b**) Intraoperative imaging after total resection of the osteophytes, allowing free range of motion

A total of four patients underwent implant revision surgery. One patient showed symptoms of impingement between the neck and cup (Fig. 1) and was therefore treated by changing the neck component from size M 15° to size M 0° 53 months after the primary surgery. In a second patient (Fig. 2), an impingement occurred between the base of the first metacarpal and remaining osteophytes between the first and second metacarpal bones, which were surgically resected and the neck component was replaced after 7 months without changing its size. Postoperatively, the symptoms completely resolved in both cases.

**Fig. 3:**
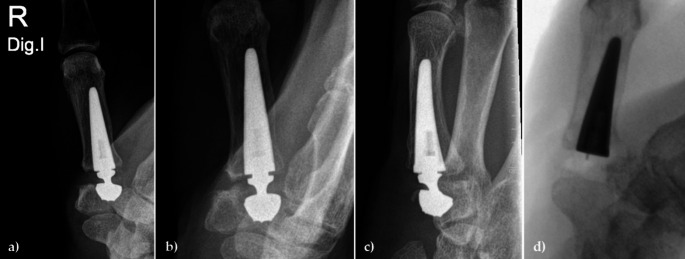
Radiographs of a patient before and after revision surgery. **a**) Radiograph after initial surgery showing primary cup misplacement; **b**) and **c**) Cup dislocation 2 years after initial surgery; **d**) Revision surgery performed as secondary trapeziectomy

**Fig. 4:**
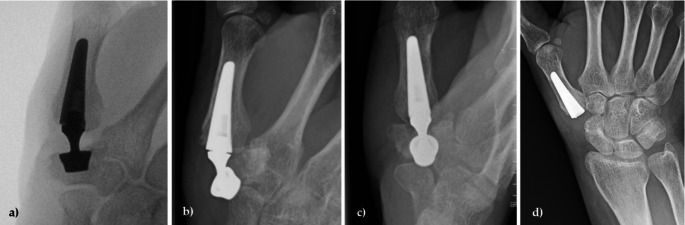
Radiographs of a patient before and after revision surgery. **a**) Intraoperative radiograph during initial surgery; **b**) and **c**) Cup dislocation 7 weeks after primary surgery due to fracture of the trapezium; **d**) Revision surgery performed as secondary trapeziectomy

Two other patients required revision due to cup dislocation (2 years and 7 weeks post-operatively). In one case, an initial cup misplacement led to the subsequent cup dislocation and revision 2 years after primary surgery (Fig. 3). The other cup dislocation at 7 weeks postoperatively was most likely the result of a trapezium fracture during the initial surgery due to multiple K-wire penetrations of the PAST (proximal articular surface of the trapezium) (Fig. 4). Both were treated by secondary trapeziectomy.

In addition, seven secondary surgeries were performed. Two patients showed clinical signs of impaired wound healing approximately two weeks after surgery and were treated with wound revision surgery, including an exchange of the mobile neck component since an infection could not be ruled out initially. Microbiological and histopathological samples were collected intraoperatively and analyzed, and no evidence of infection was found, resulting in unimpaired wound healing thereafter.

**Fig. 5:**
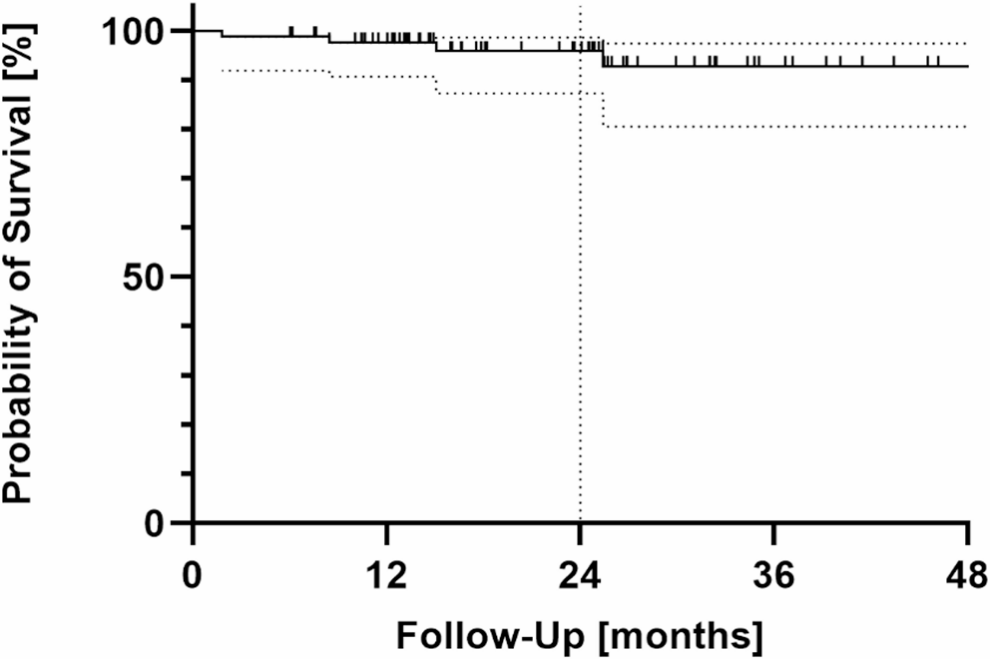
Kaplan-Meier survival analysis. The dotted vertical line represents the overall mean follow-up of 24 months. The 95% confidence intervals are shown as dotted lines above and below the Kaplan-Meier curve

Five secondary surgeries in terms of a release of the first extensor tendon compartment were performed due to secondary De Quervain tenosynovitis after an average of 10 months (range: 2.3–15.8). No other complications such as intraprosthetic dislocation or loosening of components were observed. After the mean follow-up of 2 years, the Kaplan-Meier cumulative survival rate for the endpoint of revision surgery was 96% (95% CI: 86,6–98,6) (see Fig. 5).

### Functional results

Postoperative pain levels demonstrated significant and sustained improvement compared to preoperative assessments as measured by a Numeric Rating Scale (NRS) from 1 to 10. Preoperatively, pain was notably severe under load (mean 7.5, 95% CI 6.1–8.9) and substantial even at rest (mean 4.3, 95% CI 3.8–4.9). Pain levels improved significantly after surgery with a mean NRS of 1.5 after six months (at rest: mean 1.5, 95% CI 0.3–2.7, *p* = 0.0003; under load: mean 2.6, 95% CI 0.6–4.6, *p* = 0.0004), and this improvement was consistently maintained throughout the follow-up period (*p* < 0.0001).

Thumb opposition was nearly completely restored following surgery, with sustained excellent outcomes during follow-up. The mean preoperative Kapandji score of 8.7 (95% CI 8.3–9.1) improved to 9.5–9.7 postoperatively and remained stable at 6 months (mean 9.5, 95% CI 9.2–9.7, *p* = 0.0004) and 24 months (mean 9.6, *p* < 0.0001 across 12- and 24-month follow-ups). Palmar abduction also improved significantly, increasing from a preoperative mean of 61.3° (95% CI 56.7–66.0) to 72.5° (95% CI 67.8–77.2, *p* = 0.001) at 12 months and 67.6° (95% CI 63.3–72.0, *p* = 0.046) at 24 months. Active radial abduction exhibited an improving trend, though these changes did not reach statistical significance.

Both grip and pinch strength displayed initial improvements, with significant gains at the 6-month follow-up (grip strength: preoperative mean 16.1, 95% CI 13.7–18.4; postoperative mean 22.9, 95% CI 19.1–26.7, *p* = 0.003; pinch strength: preoperative mean 4.6, 95% CI 3.9–5.2; postoperative mean 6.0, 95% CI 4.9–7.2, *p* = 0.0275). However, these improvements were not sustained at subsequent annual follow-up evaluations.

Patient-reported outcomes including the QuickDASH score and its subdomains (work and sport/music) as well as the brief Michigan Hand Questionnaire (MHQ), demonstrated significant and robust improvements postoperatively, indicating overall enhanced hand function throughout the observation period. Patient satisfaction, measured on a 1–5 scale, was consistently high, with mean scores of 4.7 at 6 and 12 months and 4.8 at 24 months, showing no significant change over time.

**Fig. 6:**
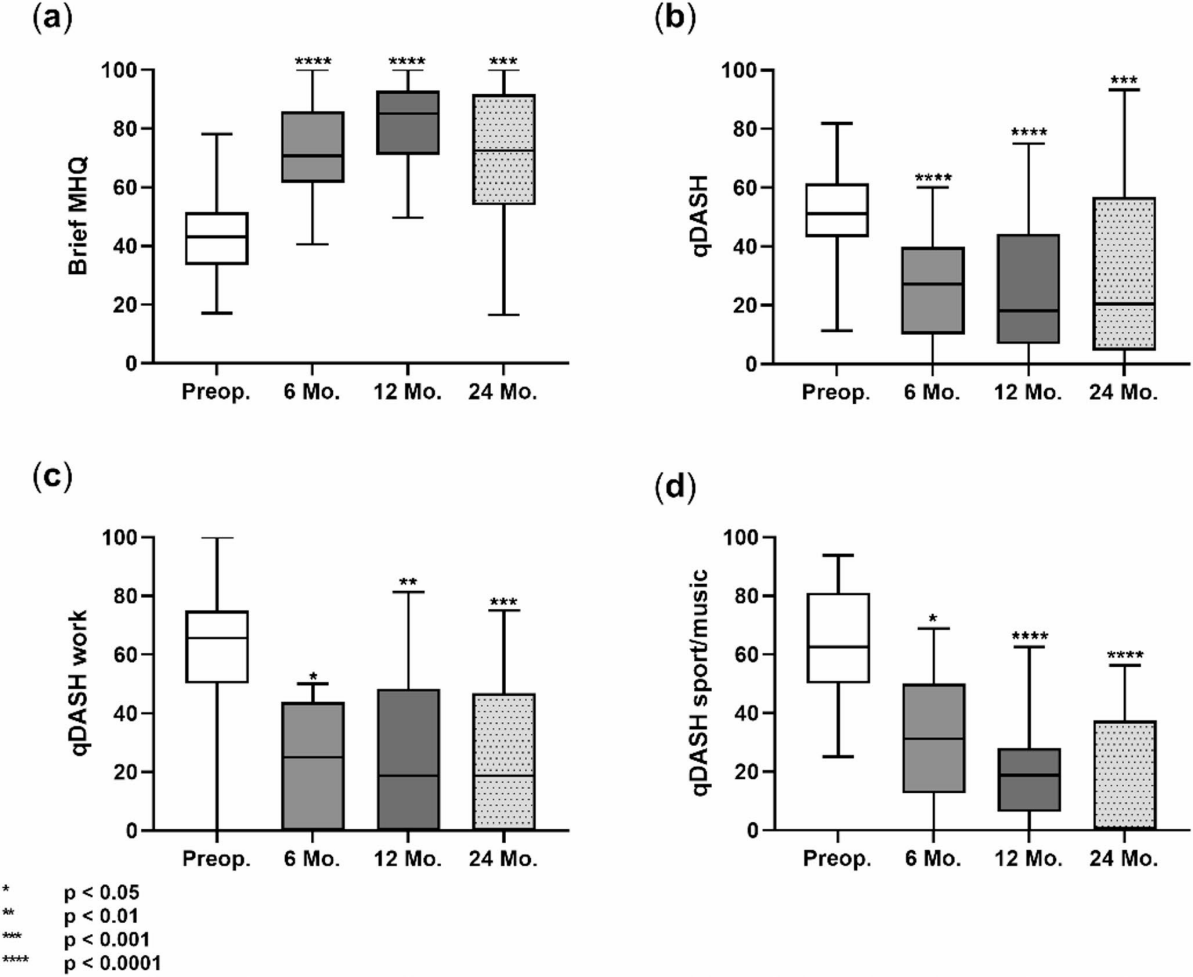
Graphical overview of functional outcome as determined by scores with box plots. **a**) Brief MHQ; **b**) QuickDASH; **c**) QuickDASH work subscore; **d**) QuickDASH sport/music subscore. Comparisons were made between preoperative and respective postoperative assessments

A summary of mean values with 95% confidence intervals for pain, range of motion (ROM), strength, and functional scores preoperatively and during follow-ups is provided in Table 1 and illustrated in Fig. 6.

**Table 1:** Summary of clinical results. Mean and 95% confidence intervals (CI) are shown below

Criteria [mean (95% CI)]	Preoperative	6 months	12 months	24 months
Pain at rest, NRS 1–10	4.3 (3.8–4.9)	1.5 (0.3–2.7) *p* = 0.0003	1.4 (1.0-1.9) *p* < 0.0001	1.5 (0.9–2.2) *p* < 0.0001
Pain under load, NRS 1–10	7.5 (6.1–8.9)	2.6 (0.6–4.6) *p* = 0.0004	3.1 (2.1-4.0) *p* < 0.0001	2.4 (1.4–3.3) *p* < 0.0001
Grip strength, kg	16.1 (13.7–18.4)	22.9 (19.1–26.7) *p* = 0.003	17.0 (14.4–19.6) *p* = 0.588 (ns)	16.0 (13.1–18.8) *p* = 0.955 (ns)
Pinch strength, kg	4.6 (3.9–5.2)	6.0 (4.9–7.2) *p* = 0.0275	5.1 (4.4–5.9) *p* = 0.281 (ns)	5.3 (4.6–5.9) *p* = 0.127 (ns)
Active radial abduction	62.6 (58.8–66.4)	63.8 (55.9–71.6) *p* = 0.8604	68.3 (63.1–73.5) *p* = 0.0968	65.6 (60.8–70.4) *p* = 0.3880
Active palmar abduction	61.3 (56.7–66.0)	64.1 (55.9–72.2) *p* = 0.5443 (ns)	72.5 (67.8–77.2) *p* = 0.0010	67.6 (63.3–72.0) *p* = 0.0460
Kapandji opposition	8.7 (8.3–9.1)	9.5 (9.2–9.7) *p* = 0.0004	9.7 (9.6–9.9) *p* < 0.0001	9.6 (9.3–9.9) *p* < 0.0001
Patient satisfaction, 1–5		4.7 (4.0-5.4)	4.7 (4.4–5.1)	4.8 (4.4–5.1)
qDASH	51.4 (47.2- 55.5)	27.1 (19.0-35.3) *p* < 0.0001	26.0 (18.7–33.3) *p* < 0.0001	29.6 (19.5–39.7) *p* = 0.0002
qDASH Work	58.1	22.5 (-5.3-50.3) *p* = 0.0178	25.0 (7.1–42.9) *p* = 0.0032	23.5 (10.3–36.8) *p* = 0.0003
qDASH Sport/Music	62.5 (51.9–73.2)	32.2 (10.9–53.4) *p* = 0,0124	21.0 (11.3–30.7) *p* < 0.0001	15.4 (4.7–26.2) *p* < 0.0001
Brief MHQ	43.5 (40.0–47.0)	71.0 (61.0-81.1) *p* < 0.0001	81.4 (74.7–88.1) *p* < 0.0001	71.8 (60.0-83.7) *p* < 0.0001

p-values comparing preoperative to postoperative measurements at the respective follow-ups were calculated with a t-test

### Radiological results

**Fig. 7:**
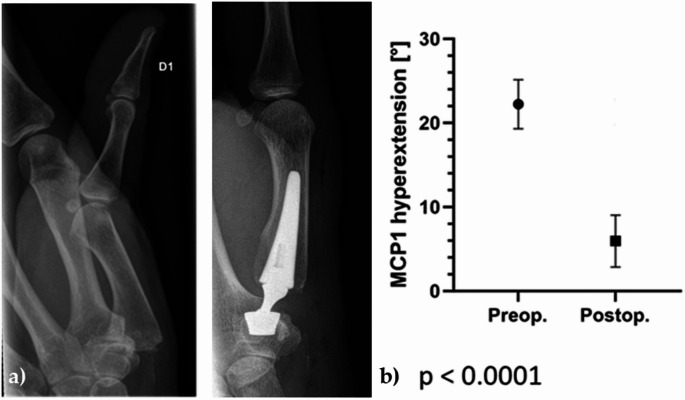
**a**) Example of metacarpophalangeal hyperextension > 15° before surgery. After implantation of the Touch® prosthesis, the deformity regressed completely; **b**) Preoperative and postoperative metacarpophalangeal (MCP) joint hyperextension before and after surgery

Analysis of preoperative radiographs showed that all patients had moderate to advanced stages of osteoarthritis (Eaton and Littler stage II or III). No patient who received a prosthesis showed evidence of significant STT osteoarthritis on pre- or post-operative radiographs. A relevant preoperative metacarpophalangeal hyperextension of > 15° in 23 thumbs (mean value preoperative 22° (range: 15–38°)) was corrected by surgery to an average of 6° (*p* < 0.0001) after 12 months (range: 0–20°) (see Fig. 7).

**Fig. 8:**
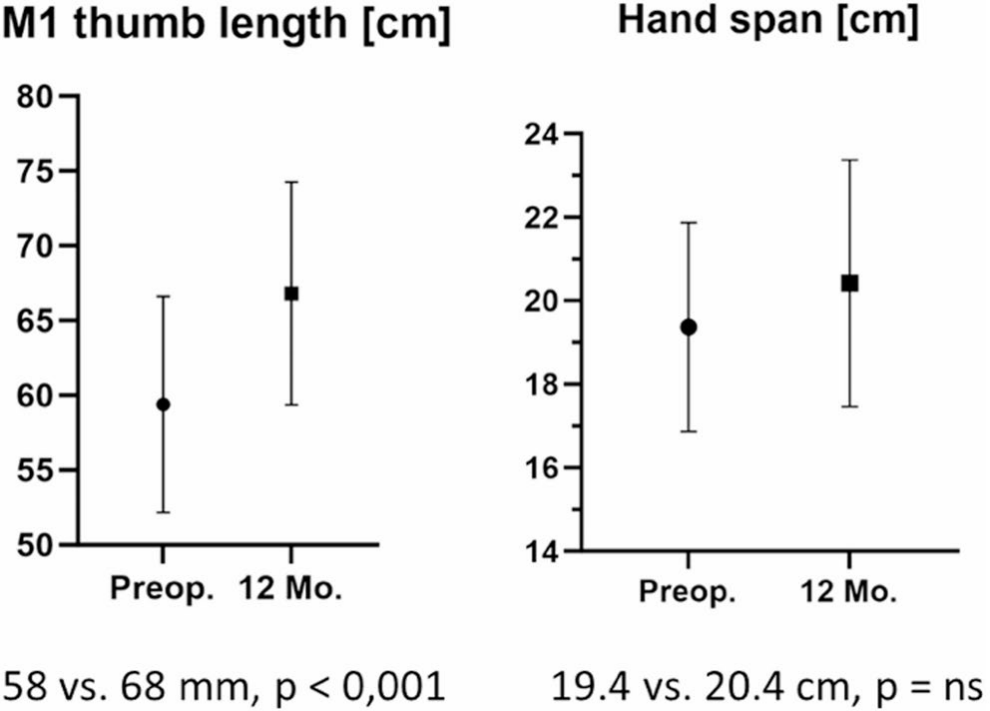
Preoperative and postoperative M1 thumb length and hand span before and one year after surgery

Postoperative radiographs showed adequate restoration of thumb length as measured by mean first metacarpal trapezium length (M1: 58 to 68 mm 12 months postoperatively, *p* < 0.001), which correlated with clinical improvements in hand span (19.4 to 20.4 cm, p = ns) (see Fig. 8).

Postoperative radiographs confirmed the adverse events described in subsection Survival, which were managed with revision surgery. No additional relevant findings such as loosening, implant dislocation or signs of inlay wear were observed.

## Discussion

This retrospective, monocentric cohort study corroborates existing evidence that dual-mobility trapeziometacarpal (TMC) prostheses effectively alleviate pain and enhance hand function in patients with advanced-stage osteoarthritis (Eaton and Littler stages II and III). Our findings demonstrate consistent improvements in pain, range of motion (ROM) and patient satisfaction, which align well with recent literature on dual-mobility implants.

To summarize our findings and experience as well as the current literature, we would like to present the key surgical steps that should be considered before, during and after the implantation of a trapeziometacarpal joint prosthesis (Table 2). These aspects were discussed in detail at the first German-speaking user meeting of the Touch Prosthesis in 2023, as published by Herren et al. [28], and have been routinely incorporated and further developed into our standard workflow in our clinic.

**Table 2:** Key surgical steps

Preoperative Planning [17, 36]	• Standard PA and lateral radiographs are sufficient in most cases. • Evaluate STT. • CT-based planning is recommended for complex as well as unclear cases and in the absence of adequate x-ray imaging: o Surgical landmarks, cysts, heights and shape (especially of the trapezium): DAST (distal articular surface of the trapezium) might be dysplastic; PAST (proximal articulating surface of the trapezium) and TRAST (trapezoidal articulating surface of the trapezium)
Surgical Approach	• Dorso-radial approach. • Protect superficial radial nerve branches and radial artery.
Handling of the Joint Capsule [44]	• Either complete capsulectomy or capsular flap preparation with closure for soft tissue barrier over implant (infection prophylaxis).
Soft Tissue and Joint Release	• Thorough release at MC1 base: o Joint capsule o Intermetacarpal ligament o Volar beak of MC1 base o Osteophytes
Preparation of First Metacarpal (MC1) [19]	• Opening of the shaft: central and not too dorsal (avoid dorsal thinning of the cortex and misalignment of the stem). • Cortical contact of the stem is not needed for primary stability. • Keep trial stem or definitive stem in place during cup preparation to protect posterior cortex of the stem.
Trapezium (Cup) Preparation [12]	• Removal of osteophytes and preservation of subchondral bone ◊ required for press-fit implantation. • Insert the guide wire perpendicular to the PAST in both lateral and dorsopalmar views. • Despite correct wire placement, the cup may be misplaced (e.g. levering of the flexible guide wire, deflection of the reamer due to sclerotic bone or inadequate release of the MC1 base). • Reaming over the K-wire manually or better with a machine (preserve bone debris). • Consider filling trapezial cysts with autologous bone or bone debris preserved from reaming.
Cup Placement	• Most critical step of the procedure • Must be: o Centered on distal trapezial surface (DAST) o Parallel to PAST • Conical cup – higher bone contact area, cannot be reoriented, no secondary tilting. • Spherical cup – less bone contact area, slightly adjustable after insertion, secondary tilting possible.
Common Pitfalls & Intraoperative Revision Options related to the cup [8, 34]	• Size 9 conical cup tilted with consecutive impingement ◊ dislocation risk. Preferred revision option: o Reposition the guide wire and ream 1–3 mm deeper into the cancellous bone o switch to larger (size 10) conical cup o Bone grafting if needed • Misplaced cups (too far in the radioulnar/dorsopalmar direction): o Replace with larger cup (any shape) o Use deeper insertion + grafting o Conical size 10 yields highest bone contact area
Implant Testing and Final Assembly	• Assess joint stability and motion with trial necks: o 15° angled neck most commonly used o Aim: Allow ½ to 1 head size subluxation under longitudinal traction (push-pull) o Avoid over-tightening (associated with pain and stiffness) • Check for impingement: o Switch to straight neck 0° if mild impingement occurs with 15° neck
Intraoperative Imaging	• Use multiplanar fluoroscopy to confirm: o Correct guidewire placement o Cup alignment to PAST o No dorsal tilt (risk of dislocation in thumb flexion)
Post-Implantation Check	• Confirm: o Free thumb opposition o No contact between implant and adjacent structures o Proper soft tissue tension and joint stability
Postoperative Care	• No cast required • Firm dressing for one week • Full weight bearing after 6 weeks

While we did not observe significant improvements in radial or palmar abduction, the total active range of motion in our cohort was above 60°, placing it in the upper normal range [2, 27] even before surgery. By comparison, Lussiez et al. reported an increase in radial abduction from 29° to 42° in their study, which remains lower than the preoperative values in our cohort [39]. However, as these measurements can depend on the examiner, direct comparisons between studies should be interpreted cautiously.

Functional analyses revealed that improvements in mean pinch and grip strength, which were significant at the 6-month follow-up, were not sustained at the annual assessments. This may reflect the heterogeneity of our patient cohort, which introduces considerable inter-individual variation. For example, grip strength in our cohort ranged from 0 to 40 kg (SD = 9.2) preoperatively and from 0 to 34.7 kg (SD = 9.4) at the 1-year follow-up, highlighting the variability in outcomes.

A study of hand function in women aged 60–69 years in Norway reported a mean DASH score of 18 [1], while Jester et al. [32] found a mean DASH score of 19.0 ± 18 in the 50–65 age group of the working population in Germany. With mean QuickDASH scores below 30 at all follow-up visits, our findings suggest a near complete recovery of hand function in relation to age. Studies reporting outcomes for the Touch® prosthesis have shown slightly better QuickDASH scores of 14 (range 6–28) [22] and 20 [39] after surgery, but these studies also reported much lower preoperative QuickDASH scores of 33 and 38, respectively, compared to a mean of 51 in our cohort.

Falkner et al. reported a mean postoperative MHQ score of 82 (range 67–92) [22] at 1 year, which is comparable to our results at 24 months and slightly better than our outcomes at 12 months. However, their preoperative MHQ scores indicate a significantly better baseline hand function (just below 70) compared to our cohort’s mean of 43.5. This discrepancy may reflect stricter surgical indications at our center or a greater willingness among our study population to tolerate impairment before seeking surgical intervention.

When comparing resection arthroplasty with joint replacement, a 3-year non-randomized prospective follow-up study suggests that both procedures result in high patient satisfaction, but joint replacement results in superior pinch strength and accelerated recovery [21]. A small study of 14 female patients with a resection-suspension-interposition arthroplasty in one thumb and an endoprosthesis in the contralateral thumb found that they were satisfied with both procedures, but the majority would prefer an implant arthroplasty, with significantly better brief MHQ scores as well as grip and pinch strength at follow-up [42].

With a cumulative survival rate of 96% for the endpoint of revision surgery at two years, the short-term results indicate that this method is both safe and associated with a low revision rate. These findings are consistent with recent literature. Holme et al. (2021) reported failure rates ranging from 2.6 to 19.9% across various implant types in a systematic review [30]. Similarly, Dremstrup observed a cumulative survival rate of 97% for 200 Moovis dual-mobility prostheses at two years, closely aligning with our results [15].

Notably, secondary trapeziectomy was required in only two of the four revised patients. In one revision, the neck component was changed from size M 15° to M 0°. As the collar of the angulated neck is wider, a slight impingement may have occurred in combination with the 15° angulation. Due to the narrower collar of the straight neck, the impingement disappeared completely after the component exchange. In cases where a tilting of the spherical cup with an impingement of the 15° angled neck has been observed, the component exchange to a straight neck as well as the repositioning of the cup have been previously described as an option of intraoperative revision during the first German-Speaking user meeting by Herren et al. [28].

In one patient who underwent revision for impingement seven months postoperatively, osteophytes were identified between the trapezium and the first metacarpal that had not been addressed at the time of initial surgery and were likely contributing to the impingement. It presented clinically as limited adduction and clicking phenomena (Fig. 2). Meticulous resection of the osteophytes between the trapezium and the first and second metacarpal bones resulted in free and unimpeded movement of the prosthesis and, therefore, a change in the size of the neck component was not necessary. In both cases we observed complete resolution of symptoms postoperatively. The learning curve must be considered an important aspect of correctly identifying these surgical challenges, especially when planning the operation, taking into account radiographs and pre- and intra-operative assessment of the size and shape of the trapezium.

To mitigate such problems, thorough resection of all osteophytes and the volar beak fragment at the base of the first metacarpal is strongly recommended. This step not only reduces the risk of impingement, but also improves surgical exposure [20]. If impingement is identified during primary surgery and cannot be resolved by an exchange of the neck component, intraoperative revision of the cup component may be required as described below [34]. Notably, as we routinely perform primary capsular release, postoperative capsule shrinkage has not been observed as a cause of reduced range of motion [51].

Further evaluation of the two patients requiring secondary trapeziectomy highlighted intraoperative challenges that, with current knowledge, could have been managed differently. Retrospective analysis suggests that primary misplacement of the cup component (Fig. 3) may have led to biomechanical complications and subsequent adverse outcomes. Correct guidewire positioning as assessed by radiographs in 2 planes is one of the most important aspects to ensure correct implantation of the cup component [12]. According to current recommendations, the cup component should be positioned at a 7° flexion angle relative to the proximal surface in lateral view and aligned parallel to the proximal and distal articular surfaces of the trapezium in cases of normal anatomy [9, 18]. With expanding knowledge and based on computer-simulated biomechanical studies [34], we would now perform an intraoperative cup revision with centralization of the component. The cup would be placed deeper in the trapezium, replaced with a size 10 conical or spherical cup, and any defect filled with cancellous bone. Postoperative immobilization for 6 weeks would then be performed to allow for adequate bone healing. It should also be noted that malpositioning of the cup with a flexion angle of more than 7° relative to the proximal surface can be corrected when using a spherical cup, but not after primary implantation of a conical cup [34]. If a secondary trapeziectomy is required, removal of the stem is not usually necessary as its complete osseointegration is usually observed. We would only remove the stem if it protruded significantly beyond the base of the first metacarpal or if there was a suspicion of infection.

In the patient who required a secondary trapeziectomy following a fracture of the trapezium with associated cup displacement, the intraoperative documentation highlighted a very soft bone structure. Additionally, the K-wire used for cup placement had to be repositioned multiple times, penetrating the scapho-trapezio-trapezoidal (STT) joint, which could have weakened the cortical bone. In conclusion, the decision to implant a prosthesis in this case represented a borderline indication. Based on this experience, we now adopt a more cautious approach to K-wire placement, ensuring it protrudes not too much into the cancellous bone before confirming its correct position with fluoroscopy. In the case of an intraoperative trapezium fracture, Herren et al. suggest osteosynthesis, e.g. with a headless screw, and postoperative immobilization for 6 weeks as a salvage and important trapezium-preserving option [28].

Despite these challenges, secondary trapeziectomy remains a reliable salvage procedure after implant failure, with outcomes comparable to those of primary trapeziectomy [10, 33, 38]. Notably, while polyethylene damage has occasionally been reported in dual-mobility prostheses, we did not observe any evidence of such damage during our revision surgeries [6].

In our cohort, De Quervain tenosynovitis occurred in 5.7% of cases and was managed either conservatively or surgically. This complication is well-documented in the literature, with reported incidences ranging from 4 to 17% following trapeziometacarpal joint replacement [24, 26]. Notably, cases of De Quervain tenosynovitis were more common in patients treated early in our series. The most likely reason for this is the lengthening of the thumb. As we gained experience, we tended to allow more telescoping than was initially recommended. We also focused on closing the joint capsule, which contributes to stability. Based on our experience, we have adjusted our surgical technique and now prioritize conservative management in the remaining cases, including hand therapy and corticosteroid injections, as symptoms often resolve over time without surgical intervention.

Contrary to recommendations by Gonzalez-Espino et al. [24], we do not perform a prophylactic release of the first extensor compartment during primary surgery. This is because such a procedure requires a longer incision and carries a potential risk of injury to the superficial branch of the radial nerve [37]. Instead, we reserve surgical release for patients with preexisting De Quervain tenosynovitis or those who already developed symptoms on the contralateral side after joint replacement. Studies suggest that De Quervain tenosynovitis is often present in a subacute or occult stage prior to surgery, or may be overlooked as symptoms are attributed to the arthritis [37]. Identifying these patients would lead to improved management of the disease before symptoms occur during post-operative rehabilitation. Preoperative imaging, such as ultrasound, could improve the diagnostic workflow and guide the decision to release the first extensor compartment during primary surgery.

Two cases required secondary surgery approximately two weeks after primary surgery due to impaired wound healing. One of these patients had insulin-dependent type 2 diabetes and a known history of wound healing complications. Both reoperations were performed primarily as prophylactic measures to prevent further complications, such as periprosthetic joint infection (PJI), during the healing process. Laboratory analyses and microbiological assessments of intraoperative tissue samples revealed no evidence of infection in either case.

Drawing from our experience and existing studies on PJI in hip and knee replacement surgeries, we strongly recommend prophylactic replacement of mobile components—specifically the neck component in this context—as a precautionary measure. Importantly, no infections were observed in any patients within this cohort throughout the study period. Furthermore, although there appears to be no biomechanical advantage to closing the joint capsule [44], it may act as a mechanical barrier to prevent infection and therefore be a useful prophylactic measure.

While single-mobility implants frequently lead to adverse events such as dislocation or loosening [3, 14, 23, 47], we did not observe these complications in the follow-up period of this study. As already demonstrated by other authors, we can agree that MCP 1 hyperextension can be successfully corrected by implanting the Touch® prosthesis [22, 39].

This study has several limitations that should be acknowledged. First, although the procedure was performed by a single surgeon, ensuring consistency in surgical technique, the potential impact of the surgeon’s learning curve [16] must be taken into account. Early and later cases may differ in outcomes due to improvements in skill and surgical protocols during the study period. In fact, two of the four revisions were performed on two of the first 10 patients operated on in our clinic. Conversely, the involvement of a single surgeon enhances reliability by minimizing variability in operative methods.

The heterogeneity of the patient cohort presents another limitation. Significant variations in preoperative clinical characteristics, such as hand function and baseline strength, complicate the interpretation of results and comparisons with other studies. For example, our cohort displayed higher preoperative impairment levels compared to those reported in similar studies, which may partially explain differences in postoperative outcomes. Additionally, the large range of individual strength measurements highlights inter-individual variability, making some findings less generalizable. A matched-pair analysis of strength measurements may have provided additional insight into individual postoperative improvement. However, this was not performed in the present study and should be considered in future investigations.

Another limitation is the relatively short follow-up period with a mean duration of 24 months. While this timeframe provides valuable insights into short-term outcomes, longer follow-up is essential to evaluate the long-term durability and performance of the Touch® prosthesis. Studies on single-mobility implants have reported survival rates of up to 95% at 10 years [20, 30], emphasizing the importance of extended follow-up for dual-mobility designs to establish comparable benchmarks [46, 47].

Finally, while this study included rigorous clinical and radiological evaluations, it lacks a control group. Future studies incorporating randomized controlled trials or direct comparisons with other prosthetic designs would provide stronger evidence for the relative benefits and limitations of the Touch® prosthesis.

## Conclusions

The Touch® dual-mobility prosthesis demonstrates significant and sustained improvements in pain relief, hand function, and range of motion for advanced TMC joint osteoarthritis, with high patient satisfaction and a low complication rate. Key outcomes, including the restoration of thumb length and correction of hyperextension, highlight the prosthesis’s technical advantages.

A cumulative survival rate of 96% at two years underscores its safety and durability, with complications largely attributable to modifiable surgical factors. While further long-term studies are needed, these findings establish the Touch® prosthesis as a reliable and effective treatment option, aligning with current evidence in the field.

## Data Availability

The raw data supporting the conclusions of this article will be made available by the authors on request.

## Abbreviations

CI: Confidence interval
CMC1: First carpometacarpal
DAST: Distal articular surface of the trapezium
HA: Hydroxyapatite
M1: Mean first metacarpal-trapezium length
MC1: First metacarpal
MCP1: First metacarpophalangeal
MHQ: Michigan Hand Questionnaire
NRS: Numeric rating scale
OA: Osteoarthritis
PA: Posterior-anterior
PAST: Proximal articular surface of the trapezium
PJI: Periprosthetic joint infection
qDASH: quick Disabilities of the Arm, Shoulder and Hand questionnaire
SD: Standard deviation
STT: Scapho-trapezio-trapezoidal
TMC: Trapeziometacarpal
TRAST: Trapezoidal articulating surface of the trapezium
